# Blockade of miR-142-3p promotes anti-apoptotic and suppressive function by inducing KDM6A-mediated H3K27me3 demethylation in induced regulatory T cells

**DOI:** 10.1038/s41419-019-1565-6

**Published:** 2019-04-15

**Authors:** Ji Gao, Jian Gu, Xiongxiong Pan, Xiaojie Gan, Zheng Ju, Shaopeng Zhang, Yongxiang Xia, Ling Lu, Xuehao Wang

**Affiliations:** 1 Hepatobiliary Center, First Affiliated Hospital, Nanjing Medical University, No. 300 Guangzhou Road, Nanjing, Jiangsu Province 210029 China; 2 Department of Anesthesiology, First Affiliated Hospital, Nanjing Medical University, No. 300 Guangzhou Road, Nanjing, Jiangsu Province 210029 China

## Abstract

In vitro induced human regulatory T cells (iTregs) have in vivo therapeutic utility. MicroRNAs (miRNAs) are a family of approximately 22-nucleotide non-coding RNAs that are processed from longer precursors by the RNases Drosha and Dicer. miRNAs regulate post-transcriptional protein expression through messenger RNA destabilization or translational silencing; miR-142-3p regulates natural Treg function through autophagy. We hypothesized that this miRNA may also have an iTreg regulation function. Antagomir-mediated knockdown of miR-142-3p improved Foxp3 (forkhead box P3) expression, regulatory function, cytokine expression, and apoptosis of iTregs in vitro, with or without inflammatory cytokine stimulation. miR-142-3p knockdown increased autophagy-related protein 16-1-mediated autophagy. Target prediction and luciferase assay results indicated that miR-142-3p binds directly to lysine demethylase 6A (KDM6A), which resulted in demethylation of H3K27me3 and in turn upregulated expression of the anti-apoptotic protein Bcl-2. Based on these results, we propose a novel strategy that uses knockdown of miR-142-3p to enhance anti-apoptotic ability and function of iTregs by increasing KDM6A and Bcl-2 expression. This approach might be used as a treatment to control established chronic immune-mediated autoimmune and inflammatory diseases.

## Introduction

Regulatory T cells (Tregs) are a subpopulation of T cells indispensable for maintenance of autoimmune tolerance^1^. They have critical roles in self-reactive lymphocyte suppression and mediation of immune homeostasis. The two major classes of Tregs are the thymic-derived Tregs (tTregs) and the in vitro induced Tregs (iTregs). Both classes have the same CD4^+^CD25^+^CD127^low^ phenotype and express the transcription factor forkhead box P3 (FOXP3). Studies using preclinical models and clinical trials found that Tregs prevent autoimmune disease and graft-versus-host disease (GVHD)^2^. The shortage of tTregs impedes the development of Treg therapy^3^. Use of adoptive transfer of iTregs has the potential because they have immune regulation functions similar to tTregs^4^. However, methods to enhance iTreg proliferation ability, survival, and function remain to be developed. In this study, we found for the first time that microRNA (miRNA) enhances the anti-apoptotic ability of iTregs through the mediation of histone modification.

Histone methylation is dynamically regulated by histone methyltransferase and demethylase to maintain gene activation and gene repression^5^. Trimethylation of H3K27 and H4K20 is associated with gene repression^6^. The anti-apoptotic gene Bcl-2 is expressed in both effector T cells and Tregs and is associated with anti-apoptotic ability and cell function^7^. Bcl-2 controls cell homeostasis of mouse iTregs^8^. Inhibition of histone demethylase decreases expression of Bcl-2 by maintaining H3K27me3 in the promoter region, which results in osteoblast apoptosis^9^. Lysine demethylase 6A (KDM6A) is also known as ubiquitously transcribed X-chromosome tetratricopeptide repeat protein, which can specifically remove the methyl group from H3K27me3. KDM6A modulates T cell differentiation by modulating the methylation status of H3K27me3^10^. Therefore, we hypothesize that KDM6A can improve the biological activity of iTregs by targeting histone demethylase to regulate histone methylation.

miRNAs are a family of small non-coding RNAs that can target messenger RNA (mRNA) transcription or mediate post-transcriptional gene repression with a short seed region complementary to mRNA sequences. miRNAs positively or negatively instruct the differentiation and suppression function of iTregs^11^. miR-142-3p can negatively regulate T cell activation in patients with systemic lupus erythematosus (SLE)^12^. Knockdown of miR-142-3p results in better proliferation and immunosuppressive ability by targeting autophagy through upregulation of autophagy-related protein 16-1 (ATG16L1) in tTreg^13^. Thus, our objective was to determine whether miR-142-3p can also regulate iTreg proliferation, survival, and immunosuppression. We were the first to find that knockdown of miR-142-3p enhanced iTreg anti-apoptotic ability and suppressive function by increasing Bcl-2 expression through promoting H3K27me3 demethylation by targeting KDM6A, both in vitro and in vivo.

## Materials and methods

### Mice

NOD CRISPR Prkdc Il2r gamma (NCG) mice, highly immunodeficient mouse, were purchased from Model Animal Research Center of Nanjing University, and housed in a specific pathogen-free facility with up to 5 mice per micro-isolator cages. All the mice were female and used at 6–8 weeks. Animal protocols were approved by Nanjing Medical University.

### Cell purification and culture

Human peripheral blood (PB) leukapheresis products of volunteers were obtained from the Department of Hematology in the Affiliated Jiangning Hospital of Nanjing Medical University. Naive human PB T cells (CD4^+^CD45RA^+^) were sort purified from PB mononuclear cells (PBMCs) (Ficoll-Hypaque, Amersham Biosciences) by magnetic-activated cell sorting (MACS pro) (Miltenyi Biotec, Germany) in a two-step procedure of magnetic beads sorting.

Naive T cells were induced to iTregs with anti-CD3/CD28 mAb-coated Dynabeads (Life Technologies, Carlsbad, CA, USA) at 1:1 (cell-to-bead) ratios in the presence of tumor growth factor-β (TGF-β) (1 ng/ml)(Bio-Techne, Abingdon, OX, USA) and recombinant interleukin-2 (IL-2) (100 U/ml) (Chiron, Emeryville, CA, USA) in X-*Vivo*-15 (BioWhittaker, Walkersville, MD, USA) media supplemented with 10% fetal bovine serum (Valley Biomedical) for 72 h.

The iTregs were cultured in the same media with the addition of recombinant IL-2 (300 U/ml) at the concentration of 0.5 × 10^6^ cells/ml. IL-2 (300 U/ml) was added every 2 or 3 days. iTregs were treated with miR inhibitor or miR mimic (Ribobio Corporation, Guangzhou, China) and renewed together with IL-2 on point days. The inhibitor group was treated with miR-142-3p inhibitor (100 nM), while the mimic group was treated with miR-142-3p mimic (50 nM). Cells were collected and tested as described.

### Flow cytometry, ImageStream, and antibodies

Human-specific monoclonal antibodies used for flow cytometry included CD4 (PE-CY7), CD8 (APC), CD25 (APC-CY7), CD45RA (BV421), HLA-A2 (PE), Annexin V (Pacific blue), propidium iodide (PI), Ki67 (PE), and Bcl-2 (APC) purchased from BioLegend, and Foxp3 (APC) and CD127 (FITC) purchased from BD Pharmingen. Among them, Ki67 (PE), Bcl-2 (APC), and Foxp3 (APC) were used for intracellular flow cytometer assay, while the others needed surface staining. Tumor necrosis factor-α (TNF-α) (FITC), interferon-γ (IFN-γ) (APC), TGF-β (FITC), and IL-10 (PE), human-specific antibodies for ICFC purchased from BioLegend, were used to detect cytokine secretion in iTregs. Annexin V (PE)/PI were applied to detect the cell apoptosis of iTreg. Sample acquisition was performed by a CATON II (BD Bioscience) and data were analyzed with FlowJo VII software (TreeStar).

### Anti- and pro-apoptotic gene expression analysis

RNA was collected from cell samples using EasyPure RNA Kit Mini (TransGen Biotech; Beijing, China). Complementary DNA (cDNA) synthesis was completed as described in the TransScrip First-Strand cDNA Synthesis SuperMix (TransGen Biotech; Beijing, China). Control gene used glyceraldehyde 3-phosphate dehydrogenase, and related gene expression (Bcl-2, Mcl-1, BID, BAD, and BAX) (all from IDT, Coralville, IA, USA) were tested on an Applied Biosystems 7500 Real-Time PCR System using Fast SYBR Green Master Mix (#4385612) and Assay on Demand primer/probe Kits (Applied Biosystems, Waltham, MA, USA). The results were further analyzed to acquire the average delta CT. PCR cycling conditions were performed as follows: 94 °C for 5 s and 60 °C for 30 s, 40 cycles and then 95 °C for 10 min.

### miRNA target prediction and luciferase assay

Potential consequential pairing of target region and miRNA was predicted by miRNA prediction software microRNA (http://www.microrna.org/), TargetScan (targetscan.org), and MIRDB (http://www.mirdb.org/). Plasmid transfections for luciferase assays in 293T cells were performed with 0.1 μg of firefly luciferase plasmid with the wild-type or mutated (mut) 3′-UTR sequences of KDM6A and 0.4 μg miR-142-3p precursor or negative control precursor along with Renilla luciferase in a 24-well plate using Lipofectamine^®^ 2000 (LP2000) as described by the manufacturer. Luciferase activity was measured 48 h post transfection using the Dual Luciferase Reporter Assay System as described by the manufacturer (Promega). The relative expression of luciferase in the miRNA-NC group was homogenized to 1 in the two groups transfected with the same luciferase plasmid. The ratio of the target miRNA group to the miRNA-NC group indicates the relative expression of luciferase. Data are mean and standard deviation (SD) of separate transfections.

### Suppression assays

The suppressive function of iTreg cultured in vitro was examined with a carboxyfluorescein succinimidyl ester (CFSE) inhibition assay as previously published^14^. Briefly, purified PBMCs were labeled with CFSE (Invitrogen) and stimulated with anti-CD3 mAb-coated beads (Dynal) ± cultured iTreg (1:2 to 1:16 iTregs/PBMCs). Four days later, cells were harvested and stained with CD4 and CD8 antibodies. Suppression was accounted from the Division Index (FlowJo, TreeStar). iTregs suppressed CD4 and CD8 T cell responses equivalently and only CD8 data are presented.

### Xenogeneic GVHD model

NCG mice between 6 and 8 weeks old were housed up to 5 per micro-isolator cage in a specific pathogen-free facility. On day 0, mice were irradiated with 150 cGys. Human PBMCs (10 × 10^6^) were transferred with or without iTregs (10 × 10^6^) treated or untreated. Clinical scores were accounted based on GVHD symptoms of mice recorded daily. Each mouse was weighed thrice weekly. Human PBMC and iTreg in PB were detected by flow cytometry on the specified dates.

### Pathological examination

Independent xenogeneic graft-versus-host disease (xGVHD) model experiments were performed as described above. Mice were sacrificed to obtain and make organ paraffin specimens on day 21. Hematoxylin–eosin (HE) staining on paraffin specimens was used to assess tissue damage and inflammatory changes. Immunofluorescence was performed with primary human monoclonal antibodies HLA-A2 and Foxp3, and staining with secondary antibodies Donkey anti-Mouse IgG (H + L) Highly Cross-Adsorbed Secondary Antibody Alexa Fluor 488 and Donkey anti-Goat IgG (H + L) Cross-Adsorbed Secondary Antibody Alexa Fluor 647 (all antibodies from Invitrogen), corresponding to HLA-A2 and Foxp3, respectively. The ImageJ software was used to analyze all quantitative images. To quantify the positive cells, images in the same series were changed to type of RGB stack and adjusted to the same threshold. Then, the “analyze particle” option of ImageJ was used to detect the positive fluorescence intensity, indicating the HLA-A2+ or Foxp3+ cell proportion.

### Statistical analysis

Reverse transcription-polymerase chain reaction (RT-PCR) data were analyzed using the SDS v2.3 software. Survival data were analyzed using Prism 5 (Mantel–Cox). Other data were analyzed by analysis of variance or Student’s *t* test. Probability (*P*) values ≤0.05 were considered statistically significant.

## Results

### miR-142-3p regulates the proliferation, Foxp3 expression, and function of iTregs in vitro

**Fig. 1:**
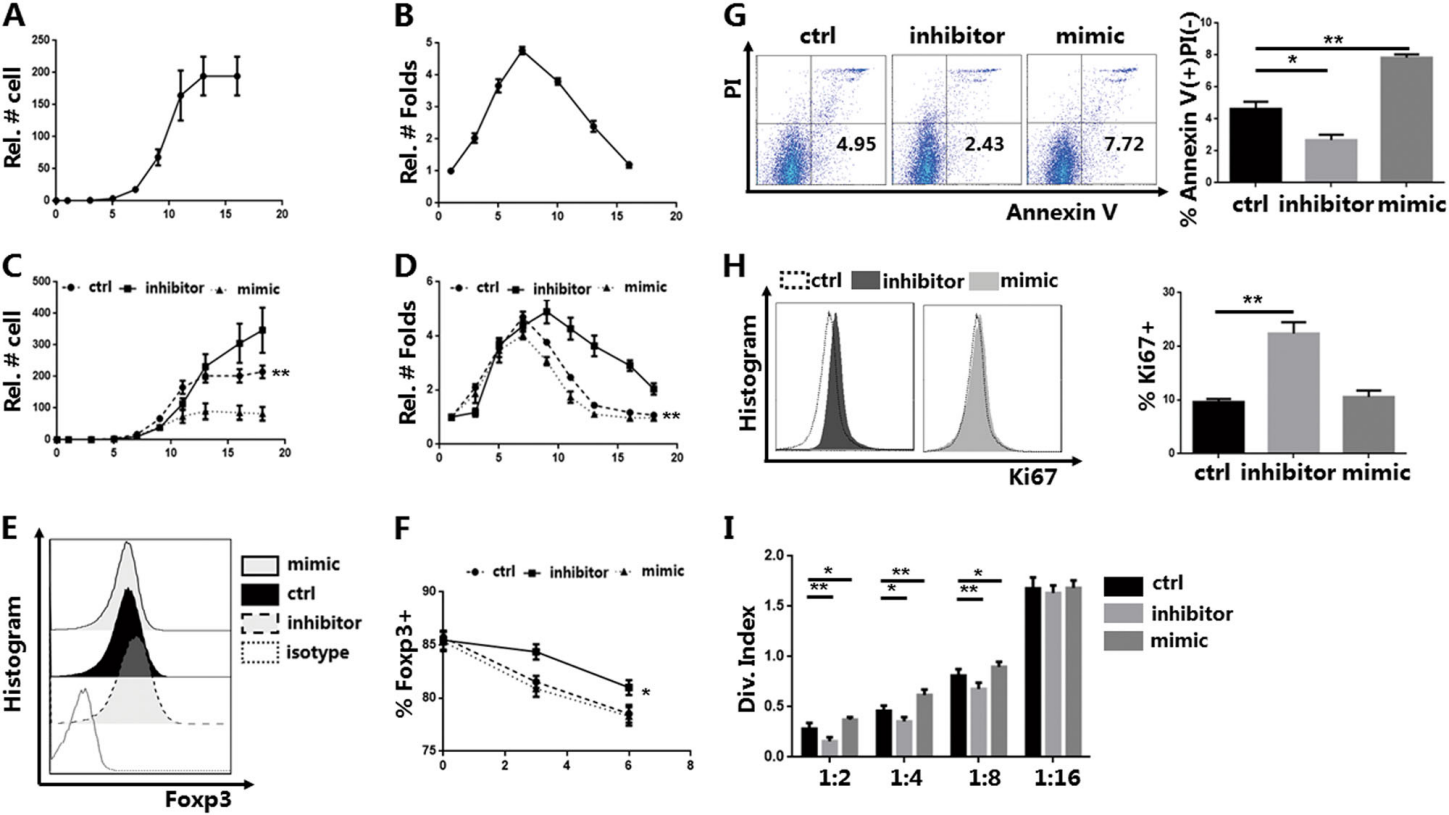
Knockdown of miR-142-3p improves the proliferation, apoptosis, and function of in vitro induced Tregs (iTregs) in vitro (*n* = 3). CD4+CD45RA+-naive T cells were sort purified using magnetic-activated cell sorting and induced as CD4+CD25+CD127−FOXP3+ iTregs, which were stimulated with anti-CD3/28 beads in the presence of fetal bovine serum (FBS) and interleukin-2 (IL-2). The iTregs were then harvested after treatment with a miR-142-3p inhibitor or mimic for 3 days on day 7. **a** Cell counts and **b** relative folds of expansion were recorded every 2 to 3 days. **c**, **d** Cell counts and relative folds of expansion of iTregs treated with miR-142-3p inhibitor or mimic were recorded every 2 to 3 days and compared to the control cells. **e** Flow detection and expression of forkhead box P3 (Foxp3) in iTregs treated with an inhibitor or mimic on day 10 (gated on CD4+CD127− cells). Representative result. **f** Foxp3 expression in iTregs of each group was recorded at different time points (days 7, 10, and 13). **g** Cell apoptosis assay using Annexin V/PI (propidium iodide) staining and % Annexin V+ PI− of iTregs from each group (gated on CD4+CD127− cells). Representative result. **h** Flow detection and positive proportion of Ki67 staining of iTregs from each group (gated on CD4+CD127− cells). Representative result. **i** Division index for CD8+ T cell mediated with anti-CD3 proliferation in vitro, at ratios from 1:2 to 1:16 (iTreg: peripheral blood mononuclear cells) as detected using carboxyfluorescein succinimidyl ester dye dilution. Mean ± standard error of the mean values are presented. *P < 0.05 and **P < 0.01

Initially, we collected CD4+CD45RA+CD25− naive T cells (purity ≥95%) from healthy donor PB samples. Naive T cells were then induced into iTregs. We observed in vitro changes in iTreg numbers and expansion for 16 days after the induction. The fold expansion reached a peak after 6–8 days of culture and then gradually decreased (Fig. 1a, b).

miR-142-3p genetic variations are associated with inflammatory bowel diseases^15^. We also previously found that miR-142-3p can negatively regulate tTreg proliferation, Foxp3 expression, and function in vitro^13^. Therefore, we also examined whether miR-142-3p controls iTreg biological properties. iTreg expansion decreased after day 7, so the inhibitor or mimic was added to the iTregs on day 7 and cell numbers and expansion were monitored. We found that knockdown of miR-142-3p promoted expansion of iTreg in vitro (Fig. 1c, d).

Next, we quantified the protein expression of Foxp3 and the suppressive function of iTregs after treatment. Knockdown of miR-142-3p using an inhibitor increased Foxp3 expression in the iTregs; treatment with a miR-142-3p mimic had no effect (Fig. 1e, f). Subsequently, we performed the suppression assay using CFSE staining. The inhibitor group suppressed CD8+ T cell proliferation via enhanced suppressive function (Fig. 1i). The mimic group did not affect Foxp3 expression. The suppressive function was downregulated, which indicated that the overexpression of miR-142-3p negatively controlled iTreg function via other mechanisms.

We also examined the expression of Ki67 to determine whether miR-142-3p controlled the proliferation of iTregs and tested cell apoptosis using Annexin-V staining with PI. After the inhibitor treatment, Ki67 expression was increased and the early apoptosis was decreased. In contrast, treatment of the mimic increased the early apoptosis of iTregs. Similar to the results for Foxp3, overexpression of miR-142-3p had no effect on Ki67 expression (Fig. 1g, h).

### Knockdown of miR-142-3p upregulates the proliferation, Foxp3 expression, and function of iTregs under inflammatory conditions

During complex disease conditions the iTreg is required to play an immunomodulatory role in the development of inflammation^16^. Therefore, we simulated the inflammatory environment in vitro by adding IL-1β and IL-6. Thus, we detected whether the iTregs were still affected by miR-142-3p under inflammatory conditions. Both IL-1β and IL-6 were added to each group for 24 h to simulate an inflammatory environment.

**Fig. 2:**
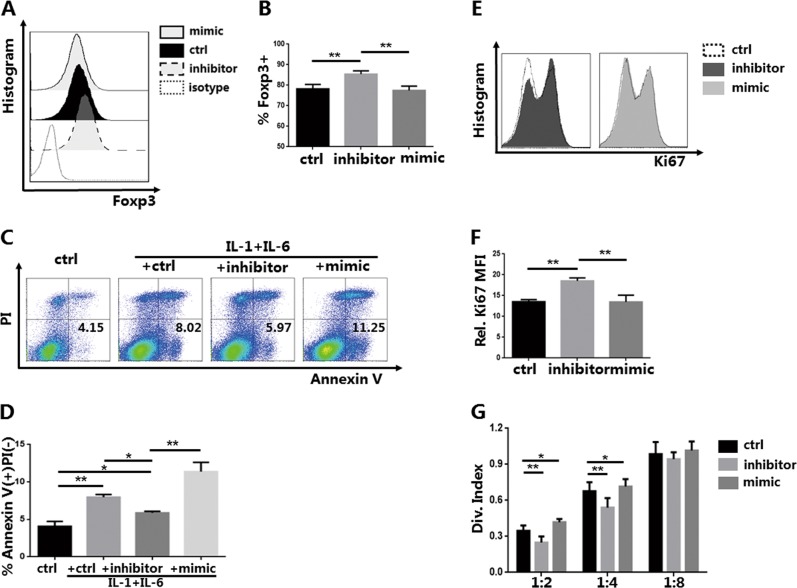
Knockdown of miR-142-3p improves the proliferation, apoptosis, and function of in vitro induced Tregs (iTregs) in an inflammatory environment (*n* = 3). Naive T cells were purified and induced as iTregs. iTregs were treated with a miR-142-3p inhibitor or mimic for 3 days on day 7. Interleukin-1β (IL-1β) (10 ng/ml) and IL-6 (10 ng/ml) (R&D Systems, Minneapolis, MN, USA) were added to the media on day 10. iTregs were harvested on day 11 for detection. **a** Forkhead box P3 (Foxp3) histogram of iTregs treated with inhibitor or mimic (gated on CD4+CD127− cells). Representative result. **b** Foxp3 expression of iTregs from each group. **c** Annexin V apoptosis detection using propidium iodide (PI) staining of iTregs treated with an inhibitor or mimic in a normal or inflammatory environment (gated on CD4+CD127− cells). Representative result. **d** %Annexin V+ PI− of iTregs from each group. **e** Ki67 histogram of iTregs treated with inhibitor or mimic in an inflammatory environment (gated on CD4+CD127− cells). Representative result. **f** Level of Ki67 expression after antagomir/agomir treatment. **g** Division index of CD8+ T cells mediated with anti-CD3 proliferation in vitro, ratio range 1:2 to 1:16 (iTreg:peripheral blood mononuclear cells) as detected using carboxyfluorescein succinimidyl ester dye dilution. Results are presented as mean ± standard error of the mean values (*P < 0.05 and **P < 0.01)

We then collected the iTregs and examined Foxp3 expression using flow cytometry. Knockdown of miR-142-3p still upregulated the expression of Foxp3; there were no changes in the mimic group (Fig. 2a, b). Concurrently, based on the results of early apoptosis measured using Annexin V/PI staining, we found that each group affected by inflammation had increased cell apoptosis (Fig. 2c, d). However, when treated using a miR-142-3p inhibitor, the increased early inflammation-induced apoptosis was attenuated; overexpression of miR-142-3p worsened the inflammation. Expression of Ki67 was promoted with a miR-142-3p inhibitor (Fig. 2e, f).

Consistent with the Foxp3 expression result, knockdown of miR-142-3p enhanced the suppressive ability of the iTregs. Similar to normal culture conditions, overexpression of miR-142-3p did not affect Foxp3 expression, but it weakened iTreg-suppressive function (Fig. 2f).

### miR-142-3p affects the secretion of anti-inflammatory and pro-inflammatory cytokines in iTregs

**Fig. 3:**
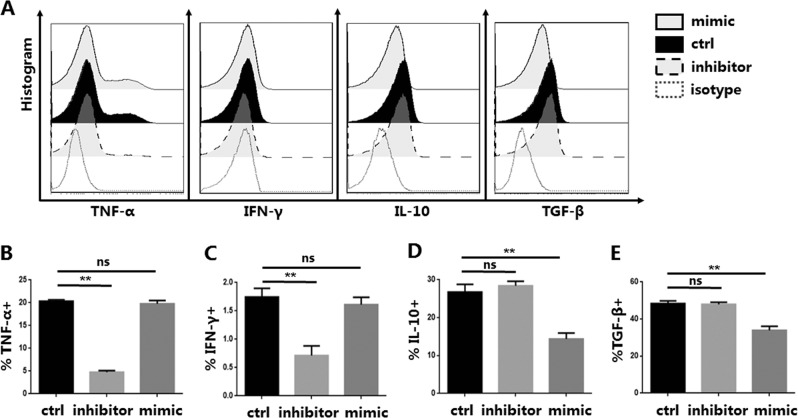
miR-142-3p affects the expression of anti-inflammatory cytokines and pro-inflammatory cytokines in in vitro induced Tregs (iTregs) (*n* = 3). iTregs were expanded in vitro and were then untreated or were incubated with an inhibitor or mimic. **a** Representative example of tumor necrosis factor-α (TNF-α), interferon-γ (IFN-γ), interleukin-10 (IL-10), and tumor growth factor-β (TGF-β) histograms for iTregs treated or not treated with the inhibitor or mimic (gated on CD4+CD127− cells). Summary of overall. **b**, and **c** Expression of TNF-α and IFN-γ was decreased in iTregs treated with the inhibitor, compared with the control. **d**, **e** Levels of IL-10 and TGF-β were decreased in iTregs treated with the mimic compared with the control. Results are presented as mean ± standard error of the mean values. **P < 0.01; ns not significant

Unlike tTregs, the mechanism by which iTregs have an immunoregulatory role is mainly through the secretion of various cytokines, in addition to the core protein Foxp3^17,18^. A series of cytokine experiments found that cytokines are associated with iTreg function and differentiation^19,20^. Therefore, we examined whether down-regulation or over-expression of miR-142-3p affected iTreg function through regulation of cytokine secretion. TGF-β and IL-10 can exert immunosuppressive effects^21^. Neither TGF-β nor IL-10 secretion changed with the miR-142-3p inhibitor treatment, compared with the control group. But overexpression of miR-142-3p resulted in significant decreases in TGF-β and IL-10 secretion (Fig. 3a, d, e).

TNF-α enhances CD8+ T cell function via activation and proliferation pathways; apoptosis and destruction of GVHD target tissue results^22–24^. IFN-γ can enhance the role of TNF-α in pro-inflammatory factors and increase TNF secretion in cultured macrophages^25,26^. TNF-α and IFN-γ did not change in the mimic group. However, knockdown of miR-142-3p significantly reduced TNF-α and IFN-γ secretion (Fig. 3b, c). Other cytokines, IL-2, IL-4, and IL-17, did not differ between the three groups (data not shown). Taken together, these results indicated that overexpression of miR-142-3p reduced the inhibitory function of iTregs by affecting cell survival and decreasing anti-inflammatory cytokine production.

### Knockdown of miR-142-3p increases ATG16L1 mRNA and ATG16L1 protein expression in iTregs

**Fig. 4:**
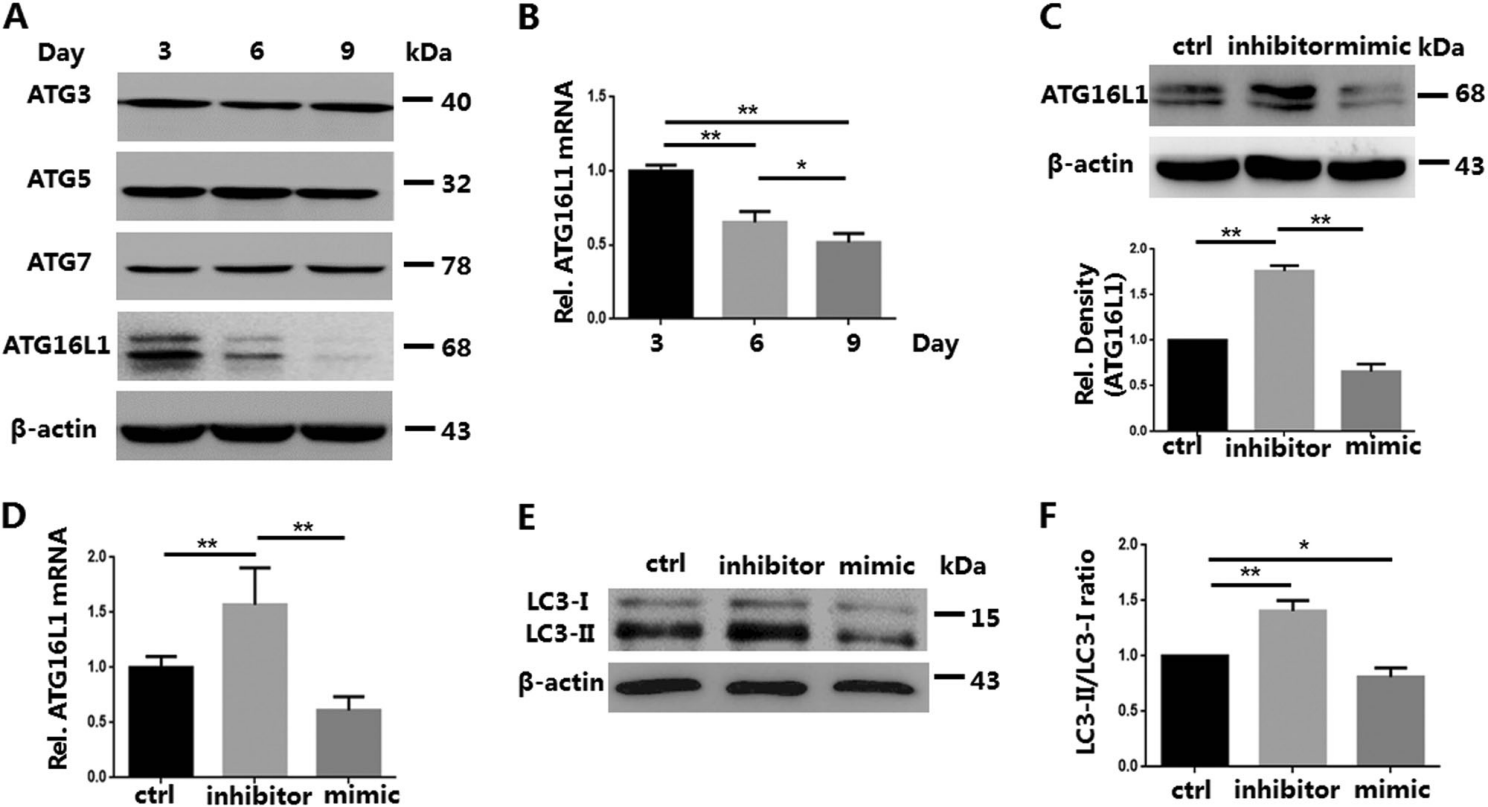
Treatment with miR-142-3p inhibitor increases the levels of autophagy-related protein 16-1 (ATG16L1) messenger RNA (mRNA) and ATG16L1 in in vitro induced Tregs (iTregs) (*n* = 3). iTregs were expanded in vitro for 7 days and then treated or not treated with the inhibitor or mimic for 3 days. Western blot and quantitative RT-PCR (qRT-PCR) were used to detect ATG expression and ATG16L1 mRNA levels of iTregs from each group, respectively. **a** ATG3/5/7/16L1 protein expression determined using western blot. **b** RNA was purified and qRT-PCR used to determine the expression of ATG16L1 mRNA at different time points (days 3, 6, and 9). **c** ATG16L1 protein levels of iTregs with or without treatment, measured using western blot. **d** RNA was purified, and qRT-PCR used to determine the expression of ATG16L1 mRNA of treated and untreated iTregs. **e** Light chain 3 (LC3) protein expression in iTregs after treatment was detected with western blot. **f** LC3-II/LC3-I ratios of three groups were analyzed. Results are presented as mean ± standard error of the mean values. *P < 0.05 and **P < 0.01

Knockdown of miR-142-3p did attenuate iTreg apoptosis. However, cell survival is associated with a variety of factors, including autophagy. Autophagy is associated with the survival and differentiation of T cells^27,28^. Our previous study found that tTreg autophagy status and ATG expression changes during the process of expansion in vitro. Therefore, we examined autophagy-related proteins associated with the survival of lymphocytes (i.e., ATG3, ATG5, ATG7, and ATG16L1)^29–32^, and only ATG16L1 decreased during iTreg expansion (Fig. 4a, b). Therefore, on day 7 we treated iTregs with an inhibitor or mimic of miR-142-3p for 3 days. Using western blot and quantitative RT-PCR (qRT-PCR), we then detected ATG16L1 protein and mRNA expression, respectively. Knockdown of miR-142-3p increased both ATG16L1 protein and mRNA expression; overexpression of miR-142-3p had an inverse effect (Fig. 4c, d). Since ATG16L1 is closely related to autophagy activity, we further used western blot to examine the expression of autophagy core protein light chain 3 (LC3) in iTreg after treatment. Consistent with ATG16L1, the ratio of LC3-II/LC3-I, which indicates cell autophagy activity, in the inhibitor group was higher than that in the other groups, while the mimic group played the opposite role (Fig. 4e, f). These results suggested that miR-142-3p may improve iTreg autophagy and Foxp3 expression through the miR-142-3p-ATG16L1-Foxp3 axis, as previously described for tTregs^13^.

### Knockdown of miR-142-3p enhances iTreg anti-apoptotic ability through promotion of demethylation of H3K27me3 via KDM6A targeting

**Fig. 5:**
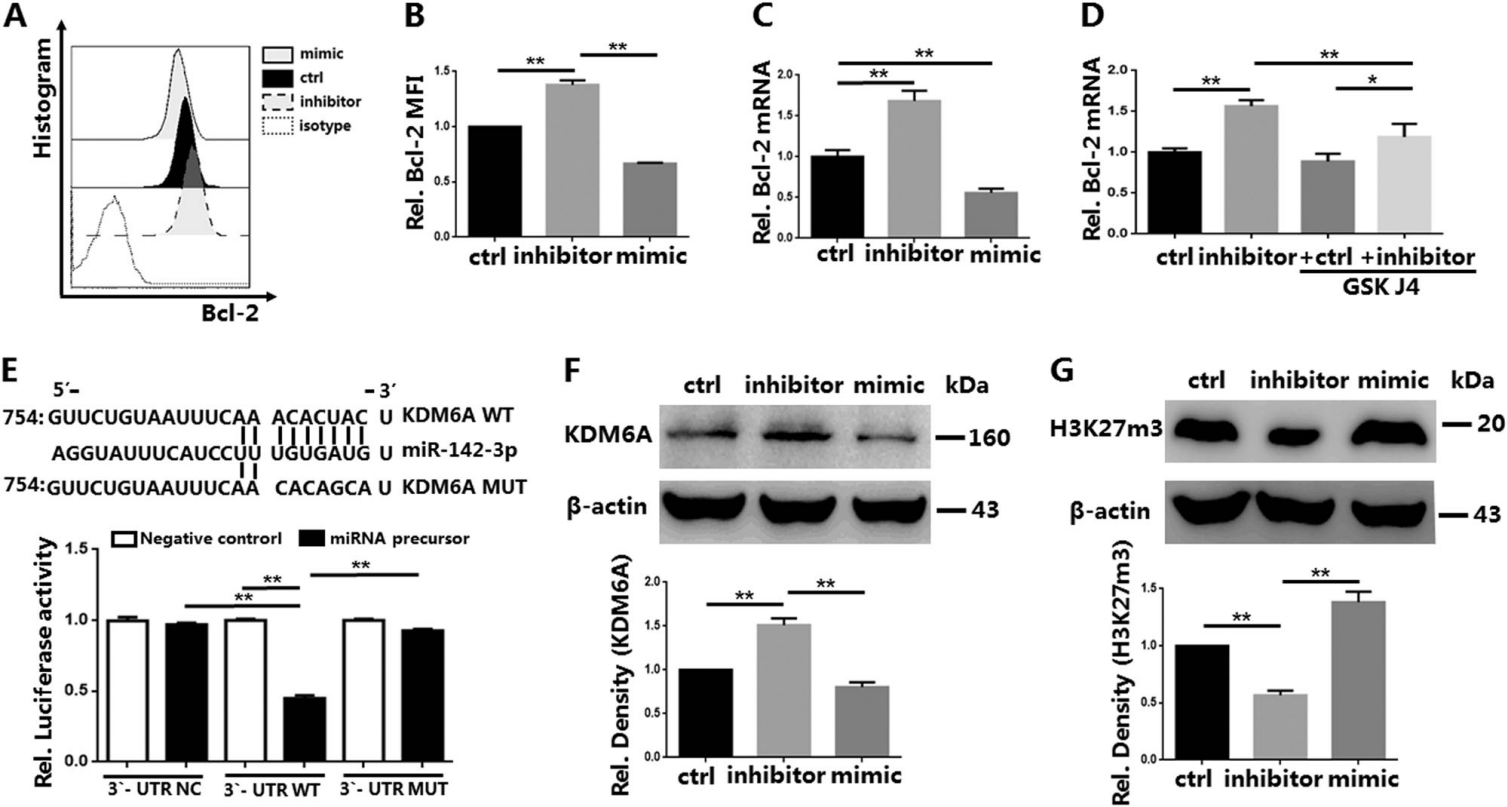
miR-142-3p decreases Bcl-2 expression and inhibits H3K27me3 demethylation by negatively regulating the target gene lysine demethylase 6A (KDM6A) in In vitro induced Tregs (iTreg) cells (*n* = 3). iTregs were expanded in vitro and treated or not treated with the inhibitor/mimic of miR-142-3p for 3 days. Following treatment, a KDM6A) inhibitor, GSK J4 (Selleckchem), was added to the control group and inhibitor group. **a** Representative example of Bcl-2 histogram for iTregs, with and without treatment. **b** Bcl-2 expression of iTregs in each group. **c** RNA was purified and quantitative RT-PCR (qRT-PCR) was used to determine the expression of Bcl-2 mRNA in each group. **d** qRT-PCR was used to determine Bcl-2 expression of the control group and inhibitor group treated or not treated with GSK J4. To determine whether human miR-142b-3p targets KDM6A mRNA, 293T cells were transduced with plasmids and luciferase reporters. **e** Schematic representation of the miR-142-3p target sequence within the 3′-UTR of KDM6A. Activity of the luciferase gene linked to the wild-type (WT) or mutant (MUT) 3′-UTR of KDM6A. Luciferase activities were measured after 48 h. The relative expression of luciferase in the miRNA-NC group was normalized to a value of 1. The results are presented as mean and standard deviation values for separate transfections (*n* = 3). KDM6A and H3K27me3 protein levels of iTregs, with and without treatment, determined using western blot (**f** and **g**, respectively). Results are presented as mean ± standard error of the mean values (*P < 0.05 and **P < 0.01)

Trimethylation of H3K27 causes gene silencing and transcriptional repression^5^. KDM6B (Jmjd3) and KDM6A are histone demethylases that can specifically promote the demethylation of H3K27me3. Bcl-2 participates in the anti-apoptotic ability and function of T lymphocytes^33,34^. Knockdown of miR-142-3p promoted the expression of Bcl-2 protein and Bcl-2 mRNA in iTregs (Fig. 5a–c). However, these changes were reversed by the addition of KDM6A inhibitor GSK J4 to the miR-142-3p inhibitor group (Fig. 5d). Therefore, we hypothesized that miR-142-3p affected H3K27 modification by targeting KDM6A, and thereby regulated Bcl-2 expression.

TargetScan (targetscan.org), MIRDB (http://www.mirdb.org/), and microRNA (http://www.microrna.org/) are the three most commonly used and most reliable miRNA target gene prediction software applications. The three software programs predicted that 6–8 nucleosides at the 5′ ends of miR-142-3P could bind to the 3′-UTR of KDM6A. The luciferase assay of KDM6A revealed the potential binding sites of miR-142-3p. miR-142-3p significantly decreased the fluorescence intensity of KDM6A, compared with the control group and the mutant group (Fig. 5e).

Western blot assay also found that miR-142-3p affected the expression of KDM6A in iTregs (Fig. 5f). We then tested whether H3K27me3 expression in iTregs changed after treatment with a miR-142-3p inhibitor. The results indicated that H3K27me3 expression was reduced in the inhibitor group (Fig. 5g). These results revealed that miR-142-3p could directly bind to KDM6A and negatively regulate KDM6A expression. Thus, knockdown of miR-142-3p promoted KDM6A expression, which resulted in increased demethylation of H3K27me3, reduced the transcriptional repression of Bcl-2, and enhanced the anti-apoptotic ability and function of iTregs.

### In an in vivo model, down-regulation of miR-142-3p significantly promotes mouse survival and inhibits GVHD progression

**Fig. 6:**
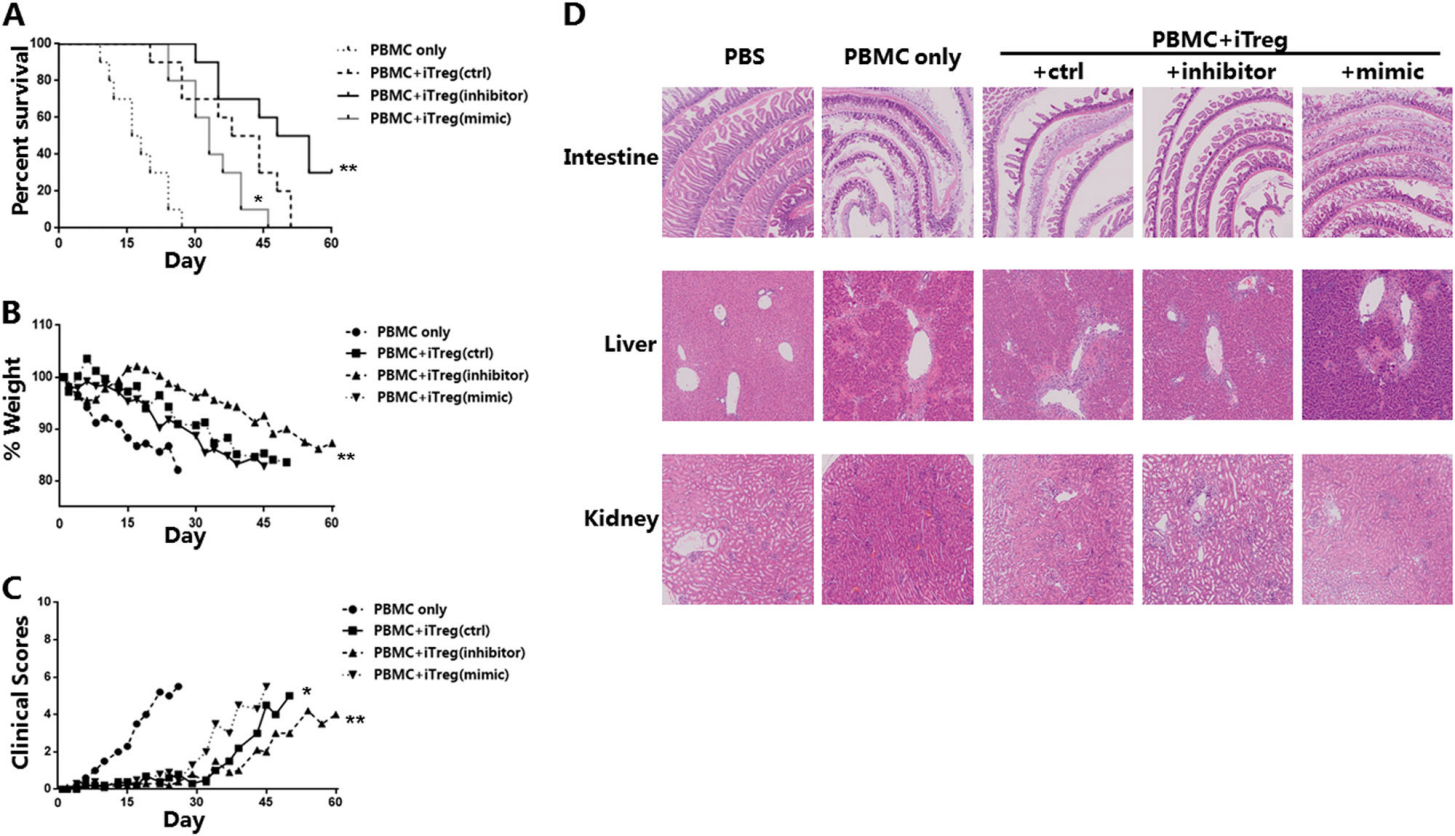
In vitro induced Tregs (iTregs) treated with miR-142-3p inhibitor results in reduced mortality and protects organs from immune damage in a xenogeneic model of graft-versus-host disease (xGVHD). Human iTregs) from volunteers were expanded in vitro and were untreated or treated with miR-142-3p inhibitor or mimic for 3 days. Following treatment, iTregs (10 × 10^6^) and allogeneic peripheral blood mononuclear cells (PBMCs) (10 × 10^6^) were washed and transferred into NOD CRISPR Prkdc Il2r gamma (NCG) mice to test the efficacy to prevent xenogeneic graft-versus-host disease (GVHD). For the groups PBMC only, control, inhibitor, and mimic, *n* = 10, 10, 10, and 10, respectively. **a** Kaplan–Meier survival curves of results when mice injected with PBMC ± groups of iTregs (*P < 0.05, **P < 0.01). **b** Average clinical scores for GVHD for mice surviving on a given day for each group (*P < 0.05, **P < 0.01 for all iTreg groups from days 0 to 60). **c** Average weight (percentage of initial) for mice surviving on a given day for each group (**P < 0.01 for all iTreg groups from days 0 to 60). **d** In another independent xGVHD experiment, mice in the four groups were humanely killed on day 21; pathological tests using hematoxylin–eosin (HE) staining of the intestine, liver, and kidney were performed for each group (*n* = 3 per group). The results shown are representative of two independent xGVHD experiments. (*P<0.05 and **P<0.01)

Knockdown of miR-142-3p promoted iTreg expansion, Foxp3 expression, and anti-apoptotic ability in vitro. To determine whether these changes improved iTreg immunosuppressive function in a GVHD model, human iTregs (10 × 10^6^) after treatment were injected with PBMC (10 × 10^6^; 1:1 ratio) into highly immunodeficient NCG mice. All three groups that received transferred iTregs had significantly reduced GVHD-induced mortality, compared with the mice receiving PBMCs only (Fig. 6a). The inhibitor group had significantly greater prolonged survival and an apparent delay in weight loss, compared with the control and mimic groups. While the mimic group had even weaker protection against GVHD (Fig. 6b).

We also evaluated a clinical indicator of GVHD in the mice; the clinical score included weight loss, activity, fur texture, posture, and skin integrity^35^. The results indicated that the inhibitor group had fewer clinical symptoms of GVHD compared with the other group (Fig. 6c). During the pathophysiological process of GVHD, multiple organs (e.g., liver, kidney, and intestine) suffer tissue damage and functional failure^36,37^. Therefore, we performed HE staining of tissues; the mice were humanely killed on day 21. The results for the mice treated with phosphate-buffered saline were normal; the other four GVHD groups had different degrees of pathological change (Fig. 6d). Severe tissue damage and numerous inflammatory cells were found in the livers, intestines, and kidneys from the PBMC group mice. Compared with the control group mice, the mimic group animals had apparent hepatocyte damage, glomerular destruction, and intestinal mucosal dissolution. These pathological changes were alleviated in the inhibitor group mice; there was some local inflammatory response, but no significant tissue damage.

### Knockdown of miR-142-3p promotes the survival and suppressive function of iTregs in vivo

**Fig. 7:**
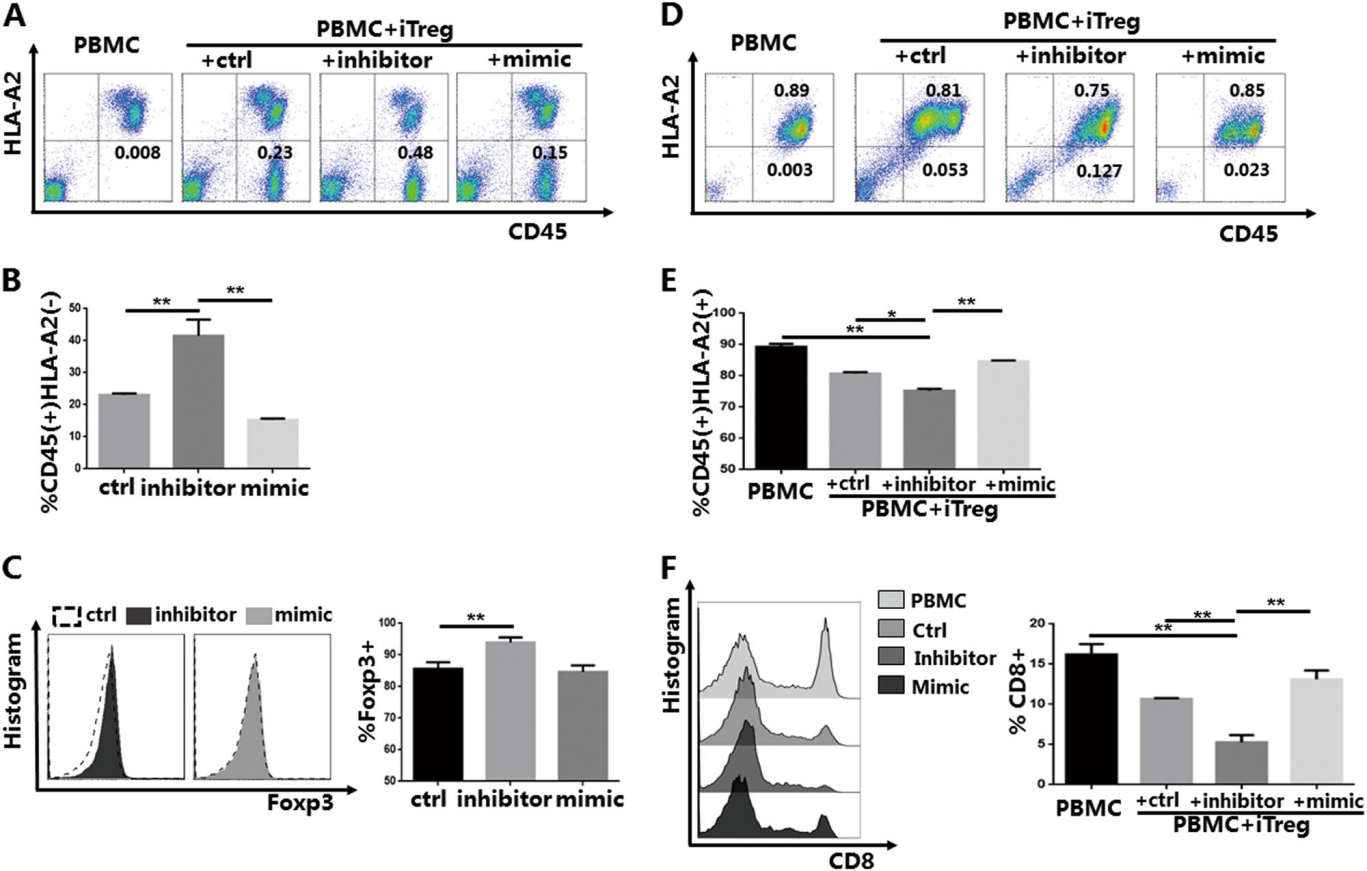
In vitro induced Tregs (iTregs) treated with miR-142-3p inhibitor have enhanced survival and function in the xenogeneic graft-versus-host disease (xGVHD) models. Peripheral blood of xGVHD mice was collected on a given day for mice surviving in each group; CD45+HLA-A2− iTreg and CD45+HLA-A2+ peripheral blood mononuclear cells (PBMCs) were then measured and recorded. **a** Representative example of CD45 versus HLA-A2 staining of blood samples collected from mice of each group on day 7 (*n* = 5) after red blood cell lysis. **b** % CD45+HLA-A2− cells for samples from each group on day 7. **c** Representative example of forkhead box P3 (Foxp3) histogram and expression of iTregs from iTreg groups on day 7 (gated on CD45+HLA-A2−CD4+ cells). **d** Representative example of CD45 versus HLA-A2 staining of blood samples collected from mice of each group on day 21 (*n* = 3) after red blood cell lysis. **e** % CD45+HLA-A2+ cells of samples from each group on day 21. **f** Representative example of CD8 histogram and % CD8+ cells of PBMCs from each group on day 21 (gated on CD45+HLA-A2+ cells). The results shown are representative of two independent xGVHD experiments. (*P<0.05 and **P<0.01)

To further examine the survival and function of iTregs after transfer into the GVHD model mice, we used the same HLA mismatch strategy used in our previous study^38^. We used HLA-A2- iTreg and HLA-A2+ PBMC mismatching to track the status of iTregs in vivo. Up to 100 μl of PB was collected from mice in the different groups on specified dates. On day 7 after infusion, the CD45+ cells in the blood samples from the PBMC-only group were almost entirely HLA-A2+ PBMCs; no HLA-A2− iTregs were detected (Fig. 7a, b). The ratio of iTregs in the inhibitor group was higher than that in the other two groups. Consistent with the in vitro results, further analysis of Foxp3 expression revealed that the inhibitor group had higher expression; there was no statistically significant difference in Foxp3 expression in the mimic group compared with the control group (Fig. 7c).

During GVHD development, CD8+ T cells have an important role in the immunopathological mechanism^39^. However, iTregs can inhibit the proliferation and function of CD8+ T cells, which is the basic principle of Treg adoptive cell therapy for GVHD^40^. The results, which are presented in Fig. 7d, indicated that CD45+HLA-A2− cells were rare in the PB of the end-stage mice in the non-inhibitor groups. There were few (12.71 ± 1.32%) cells in the inhibitor group, which indicated enhanced anti-apoptotic ability and survival. The results also indicated that the inhibitor group had fewer CD45+HLA-A2+ cells compared with the other groups (Fig. 7e). Subtype analysis of the CD45+HLAγ-A2+ cells revealed that CD8+ T cells were the most significantly suppressed cell type in the inhibitor group (Fig. 7f). The mice in the inhibitor group had the most significant and prolonged suppression of CD8+ T cells.

### miR-142-3p has effects on the infiltration of iTregs and inflammatory cells in different organs of the xenogeneic GVHD model mice

**Fig. 8:**
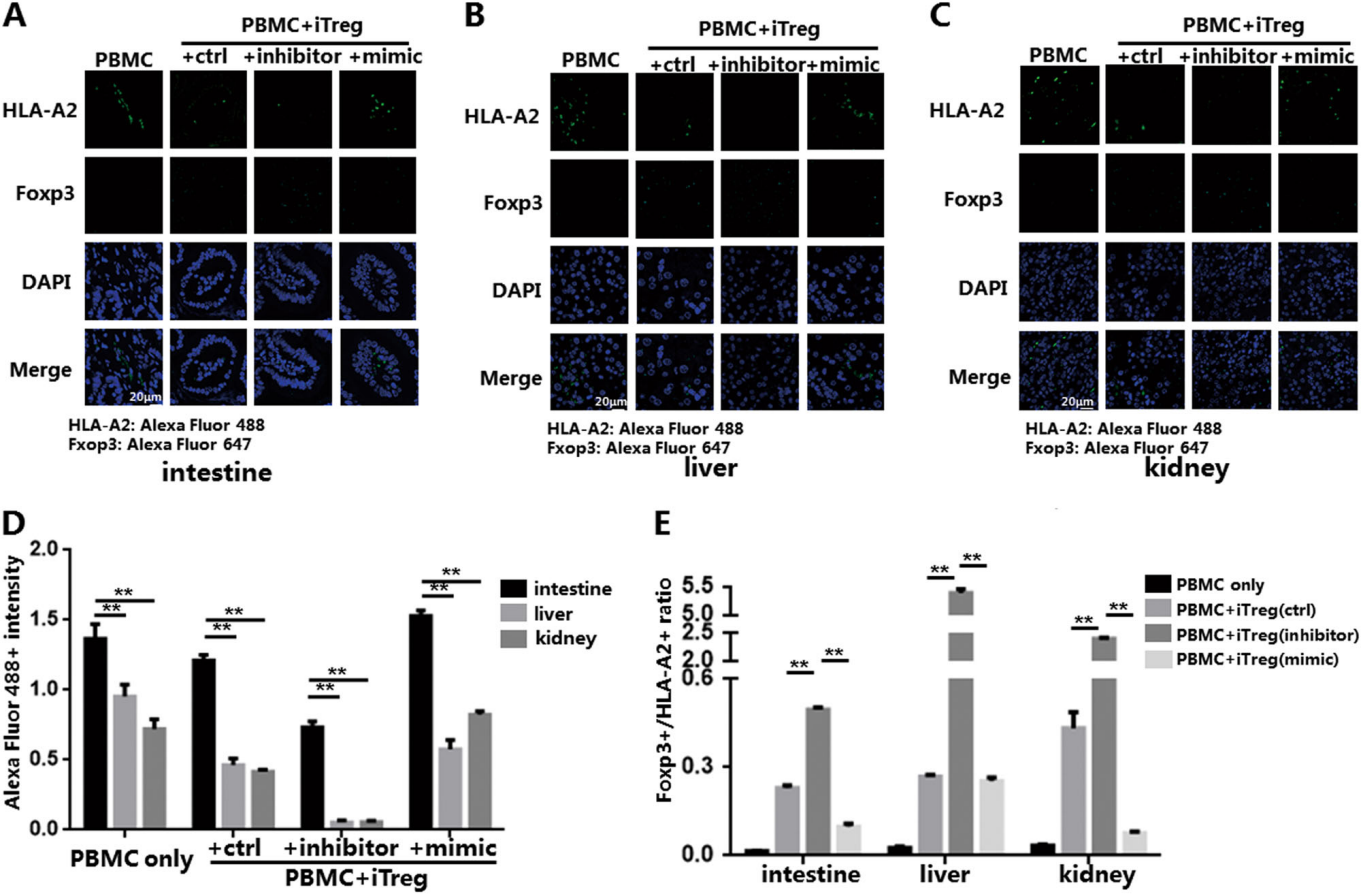
miR-142-3p affects the infiltration of in vitro induced Tregs (iTregs) and inflammatory cells in different tissues of the xenogeneic graft-versus-host disease (xGVHD) model. The paraffin-embedded specimens of mouse organs (intestine, liver, kidney) from each xGVHD group on day 21 were detected using immunofluorescence and the human monoclonal antibodies against HLA-A2 or forkhead box P3 (Foxp3) for each group; the secondary antibodies were Alexa Fluor 488 and Alexa Fluor 647. The HLA-A2+ cells and Foxp3+ cells were then measured. The positive fluorescence intensity was measured using the ImagJ software. Representative example of immunofluorescence detection results for **a** intestine, **b** liver, and **c** kidney. **d** Positive Alexa Fluor 488 fluorescence intensity of specimens from each group indicating HLA-A2+ proportion. **e** Foxp3+/ HLA-A2+ ratio of specimens from each group. Results are presented as mean ± standard error of the mean values. **P < 0.01. The results shown are representative of two independent xGVHD experiments

The HE staining results revealed that knockdown of miR-142-3p enhanced iTreg protection against immune response-associated destruction of GVHD target organs. We bound human monoclonal antibodies against HLA-A2 and Foxp3 to the secondary antibodies Alexa Fluor 488 and Alexa Fluor 647, respectively. We then detected CD45+HLA-A2+ PBMCs (Alexa Fluor 488+) and CD45+HLA-A2−Foxp3+ iTregs (Alexa Fluor 647+) in target organs on day 21 using immunofluorescence (Fig. 8a–c). Infiltration of Alexa Fluor 488+ PBMCs and Alexa Fluor 647+ iTregs differed in different groups and different target organs. Consistent with the results of other studies, we found that Alexa Fluor 488+ PBMC infiltration was most pronounced in the intestine, compared with the liver and kidney^37,41^; there were no statistically significant differences in inflammatory infiltration between liver and kidney (Fig. 8d). Compared with Alexa Fluor 488+ inflammatory cells, the number of Alexa Fluor 647+ iTregs detected in the liver and kidney was over several times greater (liver: 5.5 times; kidney: 2.5 times). Compared with the other groups, knockdown of miR-142-3p resulted in a higher Alexa Fluor 647+ iTreg proportion in all three target organs (Fig. 8e). This result indicated that knockdown of miR-142-3p enhanced the anti-apoptotic ability and survival of iTregs in vivo and that it may also affect the migration of iTregs to GVHD target organs.

## Discussion

In autoimmune diseases, self-antigen recognition disorders and dysfunction of immune tolerance results in immune attack of CD4^+^ or CD8^+^ T cells to self-antigens. This response damages target tissues^42^. The Treg is one of the immunosuppressive factors in humans, and Treg clinical trials are confronted by challenges^43^: (1) Tregs in PB (especially tTregs) are too rare to easily extract sufficient numbers, and (2) the proliferative capacity, survival, and function of Tregs need further study. miR-142-3p expression is elevated in the PB of patients with multiple sclerosis^44^. At the same time, miR-142-3p regulates the expression of intracellular ATP in regulatory T cells^45^. Therefore, miR-142-3p may be a potential target for modulating the biological activity of Treg cells.

miRNA regulates the proliferation, Foxp3 expression, and function of tTregs and iTregs through multiple pathways. For example, miR-146a decreases suppressor function by targeting Stat1 in tTregs^46^. miR-126 increases the induction and suppressive function of iTregs via the PI3K-Akt pathway^47^. Knockdown of miR-142-3p increases the expression of AC9 and cAMP in Tregs, causing decreased intracellular ATP levels, and upregulation of the functionally related protein Foxp3 expression^45,48^. miR-142-3p levels are independent of Foxp3 expression, but capable of positively or negatively regulating cell proliferation and survival^49^. However, no studies have examined the anti-apoptotic ability of human iTregs. Successful clinical application of the use of iTregs must also solve the problems associated with cell proliferation and apoptosis in iTreg culture in vitro and the resulting limited numbers of active cells^50,51^. Our study is the first to find that except for proliferation and Foxp3 expression, miR-142-3p regulated the anti-apoptotic ability of iTreg by mediating histone modification.

We found that after iTregs were cultured for 7 days in vitro, the proliferative capacity began to decrease. However, knockdown of miR-142-3p reversed these changes and increased the expression of Ki67 in the iTregs. We also found that miR-142-3p knockdown improved Foxp3 expression in iTregs; Foxp3 is the most important protein associated with the suppressor function of iTregs^52^. In addition to the core protein, Foxp3, iTreg also inhibits inflammation and immune regulation by secreting large amounts of anti-inflammation cytokines^17,18^. iTreg differentiation impaired by the increased production of TNF-α is associated with the inflammation development in experimental autoimmune encephalomyelitis^53^. TNF-α can inhibit Smad3 phosphorylation by inducing TNFR2 expression and activating AKT, resulting in repressed iTreg differentiation, decreased expression of Foxp3, and impaired function^54,55^. Foxp3+ iTreg expressed lower IFN-γ and TNF-α, compared with Foxp3^low/−^ iTreg^56^. Meanwhile, blockage of C3a/C5a reduces the secretion of IFN-γ and TNF-α in iTreg and enhanced the suppressive function in GVHD^57^. Knockdown of miR-142-3p significantly decreased the expression of TNF-α and IFN-γ. However, miR-142-3p overexpression did not promote the expression of TNF-α or IFN-γ, it repressed the expression of TGF-β and IL-10.

As an autophagy-related protein, ATG16L1 controls cell survival and function by regulating the autophagy state during the development of T lymphocytes^58^. ATG16L1 also regulates T cell activation and differentiation in some autoimmune diseases, such as inflammatory bowel disease and SLE^59,60^. Similar to the tTregs^13^, only ATG16L1 expression changed; it decreased during iTreg culture. We also found that treatment with a miR-142-3p inhibitor reversed the changes. Consistent with the change of ATG16L1, knockdown of miR-142-3p increased LC3-II/LC3-I ratio in iTreg, indicating strengthened autophagy activity. This result suggested that similar to the tTregs, knockdown of miR-142-3p regulates the function and autophagy of iTreg via the miR-142-3p–ATG16L1–Foxp3 axis.

Histone modification is an important mechanism regulating gene expression. Histone modification is also closely related to T cell proliferation and apoptosis^61^. We found that miR-142-3p regulated the methylation status of histones in iTregs. Deletion of KDM6A results in a decrease in PB T cells^62^. In multiple cell types, aggregation of H3K27me3 at the promoter of Bcl-2 induces cell apoptosis^9,63,64^. We found that miR-142-3p negatively regulated the expression of the target gene KDM6A, which in turn modulated the demethylation status of H3K27me3 in iTregs. At the same time, miR-142-3p inhibited Bcl-2 expression in the iTregs. These results indicated that we can inhibit miR-142-3p to enhance the demethylation of H3K27me3 by targeting KDM6A, thereby upregulating the expression of Bcl-2 in iTregs and enhancing the anti-apoptotic ability of iTregs, both in vivo and in vitro.

However, the clinical application of iTreg use is hindered by the challenges associated with maintaining sustained high levels of Foxp3 and Treg survival in vivo^51^. Consistent with the in vitro results, knockdown of miR-142-3p increased Foxp3 expression and survival of iTregs in vivo. As the stable survival of iTreg, both in vivo and in vitro, is essential for adoptive therapy, we found knockdown of miR-142-3p enhanced the autophagy activity and anti-apoptotic ability of iTreg, resulting in prolonged survival. This demonstrates that prolonged survival of iTreg can better exert the therapeutic effect of GVHD. Subsequently, the pathological examination that included HE staining and immunofluorescence assay revealed that knockdown of miR-142-3p enhanced the anti-apoptotic ability of iTregs, prolonged the survival in vivo, and increased protection against GVHD. miR-142-3p may also be involved in the regulation of iTreg migration.

In summary, this study is the first to find that addition of a miR-142-3p inhibitor to iTregs cultured in vitro enhanced iTreg resistance with increased anti-apoptotic ability, immunosuppressive function, and maintained Foxp3 expression both in vivo and in vitro, through the miR-142-3p–KDM6A–H3K27me3–Bcl-2 pathway. Study of the related mechanisms of miRNA and histone modification represents a new direction for iTreg clinical trials.
